# Hair-Normalized Cortisol Waking Response as a Novel Biomarker of Hypothalamic-Pituitary-Adrenal Axis Activity following Acute Trauma: A Proof-of-Concept Study with Pilot Results

**DOI:** 10.1155/2013/876871

**Published:** 2013-12-03

**Authors:** David M. Walton, Joy C. MacDermid, Evan Russell, Gideon Koren, Stan Van Uum

**Affiliations:** ^1^School of Physical Therapy, Western University, London, ON, Canada N6G 1H1; ^2^School of Physical Therapy, McMaster University, Hamilton, ON, Canada L8S 1C7; ^3^Department of Physiology and Pharmacology, Western University, London, ON, Canada N6A 5C1; ^4^Ivey Chair in Molecular Toxicology, Western University, London, ON, Canada N6A 5C1; ^5^Department of Pharmacology/Toxicology, Hospital for Sick Children, Toronto, ON, Canada M5G 1X8; ^6^Department of Medicine, Schulich School of Medicine and Dentistry, Western University, London, ON, Canada N6A 5C1

## Abstract

The mechanisms underlying the development of persistent posttraumatic pain and disability remain elusive. Recent evidence suggests that disordered stress-system pathway (hypothalamic-pituitary-adrenal axis) activity may be responsible for the genesis and maintenance of long-term sensory and emotional problems. However, confidence in current evidence is limited by the necessarily retrospective collection of data. Hair cortisol can serve as a calendar of HPA axis activity going back several months prior to injury. The purposes of this pilot study were to determine the feasibility of using hair cortisol and hair-normalized salivary cortisol as biomarkers of distress following traumatic injuries of whiplash or distal radius fracture. Ten subjects provided complete data within 3 weeks of injury. Hair cortisol, cortisol waking response (CWR), and mean daily cortisol (MDC) were captured at inception, as were self-report indicators of pain, disability, and pain catastrophizing. Pain and disability were also captured 3 months after injury. Results indicate that cortisol waking response may be a useful biomarker of current distress as measured using the pain catastrophizing scale, especially when normalized to 3-month hair cortisol (*r* = 0.77 raw, 0.93 normalized). Hair-normalized CWR may also have predictive capacity, correlating with 3-month self-reported disability at *r* = 0.70. While promising, the results must be viewed in light of the small sample.

## 1. Introduction

Chronic pain and disability are responsible for staggering socioeconomic burden [1–3], estimated to affect between 1 in 3 and 1 in 5 adults in developed countries [4, 5]. Effective treatment for noncancer chronic pain and disability remains elusive, usually relying on multimodal care often including complex and potentially risky pharmaceutical regimens. Logically, predicting and preventing the development of chronic problems could solve many of these current challenges. However, the mechanisms underlying the transition from acute to chronic pain and disability following musculoskeletal trauma remain poorly understood.

A number of theoretical models or frameworks have been proposed in an attempt to demystify the development of chronic pain. These range from purely structural/anatomical [6] to purely cognitive [7] and to integrated biological and psychological [8]. A consistent thread across many of the existing frameworks and empirical studies addressing prognosis or features of chronic pain is the presence of physiological or emotional distress. Distress is a key component of many cognitive models, often framed in terms of psychological constructs such as “fear” or “catastrophizing.” Systematic reviews or meta-analyses often find support for psychological distress as a prognostic variable for predicting the development of long-term problems following conditions such as acute whiplash [9] or low back pain [10, 11]. One clear application of such findings is the advent and proliferation of educational interventions intended to reduce distress for patients with acute injuries. Whether they are standardized (e.g., pamphlets) or informal (e.g., clinical discussion), many current best practice guidelines endorse “advice and education to stay active” and “reassurance” as first-tier therapies following acute, nonlife-threatening traumas [12]. This approach is built upon the notion that sound education and reassurance can relieve the distress experienced by the patient.

While the relationship between acute self-reported psychological distress and mid- to long-term outcome is well established, the biological mechanisms involved have received less attention. The hypothalamic-pituitary-adrenal (HPA) axis is the major physiological stress-response pathway, and its activation results in the release of the stress hormone cortisol as an adaptive strategy for dealing with potential danger, whether the danger is real or perceived. Cortisol has wide-reaching effects, including slowed digestion, tissue catabolism, disordered immune/inflammatory activity, and elevated autonomic function, all in preparation for so-called “fight or flight” reactions. The clinical presentation of exaggerated HPA axis function, including generalized pain, digestive problems, impaired sleep, and cognition [13, 14], bears striking resemblance to that of many chronic musculoskeletal pain problems, with recent evidence mounting to support a direct link with fibromyalgia [15]. HPA axis dysfunction has also been demonstrated in more common musculoskeletal pain problems such as chronic whiplash [16], chronic low back pain [17], and complex regional pain syndrome [18]. In most of these cases, HPA axis activity has been estimated using common clinical indicators of axis activity, such as the cortisol waking response (CWR), mean daily cortisol (MDC), or diurnal cortisol decline (DCD).

The effect of acute HPA axis activity on posttraumatic emotional or physiological distress and subsequent recovery has not been adequately explored to date. One potential explanation is that salivary cortisol, while easy to obtain and assay, is considerably variable across the day with wide interindividual variation, likely more so in people with acute traumatic injuries. However, advancements in cortisol assay techniques have provided a potential solution to this problem, which is the ability to assay cortisol concentrations in hair samples [19].

Throughout the past decade analysis of cortisol in hair has emerged as a new way to measure chronic stress. Cortisol is assumed to enter the hair shaft primarily through the capillaries perfusing the bulb, as well as from sebum and sweat [20]. If hair grows at an average rate of 1 cm/month with an estimated lag of 5–15 days for cortisol to enter the hair [21], each 1 centimeter distal to the human scalp represents the integral of roughly the past month's systemic cortisol secretion. This allows for retrospective examination of cortisol production over months or even years. This is highly advantageous, since one of the flaws in many of the current cohort studies is that they analyze psychological features of patients *after* the injury and attribute the psychological correlates to the results of trauma and when it is possible they preceded the injury. Hair cortisol analysis is a proven method, capable of measuring stress from a wide array of physiological, pathological, and social stressors, including stress due to chronic pain [22]. van Uum and colleagues [23] found that participants using opioids for the management of severe chronic pain had significantly higher hair cortisol and significantly higher perceived stress when compared to nonpainful age- and sex-matched controls. This study provides a strong indication that hair cortisol may be able to provide new information regarding the HPA axis activity of those who are rehabilitating following acute trauma.

We propose that through normalization to trait hair cortisol concentrations, “state” salivary cortisol will be more easily interpretable and comparable across individuals. The purpose of this proof-of-concept study was to provide pilot data on the potential value of hair-normalized salivary cortisol in comparison to self-reported measures of acute psychological distress. A secondary purpose was to determine whether a relationship appears to exist between acute hair-normalized cortisol and midterm outcomes following traumatic injury.

## 2. Methods

This was a prospective cohort study. Subjects were sampled consecutively through posters placed at local rehabilitation (physiotherapy, chiropractic) clinics or emergency departments. Subjects were eligible if they presented within 4 weeks (28 days) of a traumatic injury to either their neck as a result of a motor vehicle accident (i.e., whiplash associated disorder (WAD)) or a distal radius fracture (DRF) as a result of a fall, were between 18 and 65 years old, and could speak and understand conversational English. Subjects also had to have at least 3 cm of hair over the posterior vertex of their scalp. Subjects were excluded if they were pregnant or planning to become pregnant, had been hospitalized overnight within the previous 3 months, had taken any steroid-based medication within the previous 6 months, had suffered a concussion or closed-head injury, or had any other serious systemic (e.g., cancer, liver, heart or kidney disease, and known hyper- or hypocortisolism) or neuromuscular (e.g., stroke, multiple sclerosis) disorder. All methods were approved by the Health Sciences Research Ethics Board at Western University prior to initiating subject recruitment. All subjects gave written informed consent.

Recruitment and baseline assessment occurred as close as possible to the traumatic event. Baseline data captured included a subject characteristics form (age, sex, duration of symptoms, medicolegal and work status, other stressors experienced within the past 3 months using a checklist, and current medications), a pain intensity numeric rating scale (NRS, [24]), a region-specific disability scale: either the neck disability index (NDI, [25]) or the patient-rated wrist evaluation (PRWE, [26]), and the Pain Catastrophizing Scale (PCS, [27]).

The 0–10 NRS is the most widely used pain intensity measure and has ample evidence of sound measurement properties in these and other conditions [28]. The NDI is the most widely-used neck-specific disability scale [29] and enjoys evidence of generally sound measurement properties (reliability and validity, [30]). It is composed of 10 items that include both symptom and function-related domains, each scored between 0 and 5 where a higher score indicates greater disability. The score out of 50 can be easily converted to a score out of 100 for the purposes of comparison across scales. The PRWE possesses adequate measurement properties (reliability and validity, [26]) and is one of the only wrist-specific disability scales currently available. It is composed of two subscales, pain and function, where the total score is expressed out of 100. Both the NDI and PRWE measure similar constructs for their respective region. The PCS is the most widely-used scale for measuring pain-related catastrophic beliefs. It has consistently demonstrated sound measurement properties [31] and is a consistent predictor of postinjury recovery in both WAD [9, 32] and DRF [33]. It is composed of 13 items each rated on a scale from 0 to 4, where a higher number indicates greater catastrophic (exaggerated negative orientation) beliefs about pain.

Subjects also provided a sample of approximately 100 hairs from the posterior vertex of the scalp, harvested using sterile scissors as close to the scalp as possible. Subjects were subsequently sent home with 3 sterile Salivettes and instructed to provide 3 saliva samples on their next nonworking day: T1, immediately upon waking, T2, 30 minutes after waking, and T3, between the hours of 15:00 and 16:00 the same afternoon. They were instructed not to eat or drink and not to engage in sexual or strenuous physical activity for the hour prior to collection. Samples were immediately stored in a residential freezer until they were collected by a member of the study team and sent for assay.

Followup occurred 12 weeks following the initiating trauma. The same data were collected at followup save for the hair sample. Recovery status was assessed through an amalgam of symptom intensity (0–10 numeric rating scale), region-specific disability (NDI or PRWE, converted to percent).

### 2.1. Assay Technique

Upon obtaining the saliva samples, salivettes were centrifuged at 2218 g for 10 minutes. Following this, 500 *μ*L of saliva was pipetted out and stored in 1.5 mL Eppendorf tubes at −20°C until analysis. Prior to analysis saliva samples were allowed to thaw at room temperature for 1 hour. Analysis was performed using the commercially available salivary cortisol immunoassay (Alpco Diagnostics, Salem, NH, USA).

The most proximal 3 cm of the hair samples was sectioned and 10–15 mg of hair obtained. Hair segments were placed in 20 mL scintillation vials and washed twice for 3 minutes at 0.28 g with 3 mL of isopropanol. Following the washes, samples were allowed to dry under a fume hood for a minimum of 5 hours. The remainder of hair analysis was performed using our previously established laboratory protocol [34]. In short, 1 mL of methanol was added to each of the vials and the hair segments were minced finely with surgical scissors. The vials were then incubated at 50°C for 16 hours on an incubator shaker at 0.28 g. Subsequently, the methanol was evaporated with nitrogen gas at 50°C. The remaining residue was reconstituted with 250 *μ*L of PBS (pH 8.0) and analysis was performed on a salivary cortisol immunoassay (Alpco Diagnostics, Salem, NH, USA). Cortisol concentrations were then corrected to hair mass to get a hair cortisol concentration in ng/g. Intra- and interassay coefficients of variation were determined to be 5.7% and 11.1%, respectively.

## 3. Analysis

Subject characteristics (age, sex, and duration of symptoms) were evaluated descriptively (frequencies or means and standard deviations). Cortisol waking response (waking to 30 minutes post waking, ng/mL) and mean 1-day salivary cortisol (waking, 30 minutes after waking, midafternoon, ng/mL) were calculated for each subject. Data fidelity was determined through observation of the behaviour of salivary cortisol. Normative data on CWR or MDC in people with acute injuries was not available, but previous evidence in nonobese, healthy, adult subjects indicates that a mean CWR of between 25 and 50% is expected and may range from a high of 75% increase to a low of 10% decrease [35, 36]. For the purposes of this pilot study, those subjects who obviously deviated from the expected diurnal variation, that is, who showed dramatic negative slope (>15% change in the wrong direction) or a dramatic positive slope (>80% increase) of the cortisol waking response, were assumed to have not complied with the protocol for saliva collection, had an undisclosed condition that affected HPA activity, or experienced some external factor that influenced HPA activity (e.g., forced early waking or startled waking) and were therefore removed. Hair cortisol, mean 1-day salivary cortisol, and cortisol waking response were evaluated for normality through the Shapiro-Wilk test. Assuming acceptable normality, the ratio of mean 1-day salivary cortisol to hair cortisol (hair-normalized mean daily cortisol (HnMDC)) and cortisol waking response to hair cortisol (hair-normalized cortisol waking response (HnCWR)) were created for each subject as an indicator of state (salivary) to trait (hair) HPA activity.

Cross-sectional correlations were conducted using unadjusted CWR and MDC and both HnMDC and HnCWR at baseline as dependent variables. Independent variables were sex and age, baseline PCS, NRS, and percent disability. Mean sex differences were evaluated using independent samples *t*-test, and the correlation with continuous variables was evaluated using Pearson's *r*. Longitudinal relationships were evaluated similarly, in this case using HnMDC and HnCWR as independent (predictor) variables and 3-month percent disability and NRS as dependent variables. As an exploratory pilot study, no *a priori* hypotheses were posed.

## 4. Results

Between June 2011 and December 2012, 25 subjects were approached and 15 subjects consented to participate in this pilot study. Of those, 3 males did not have sufficient hair to conduct a valid assay of hair cortisol, 1 subject appeared to be noncompliant with the saliva collection protocol (evidenced by a 40% reduction in salivary cortisol between waking and 30 minutes after waking), and the saliva sample from 1 additional subject appeared to be contaminated (salivary cortisol > 10000 ng/mL). This left a sample of 10 subjects (7 female) with complete and valid data for analysis.  Table 1 presents the characteristics of the 10 subjects and descriptive statistics for each of the dependent and independent variables. Normality of the primary variables (HnMDC and HnCWR) was confirmed through nonsignificant Shapiro-Wilk tests.

**Figure 1:**
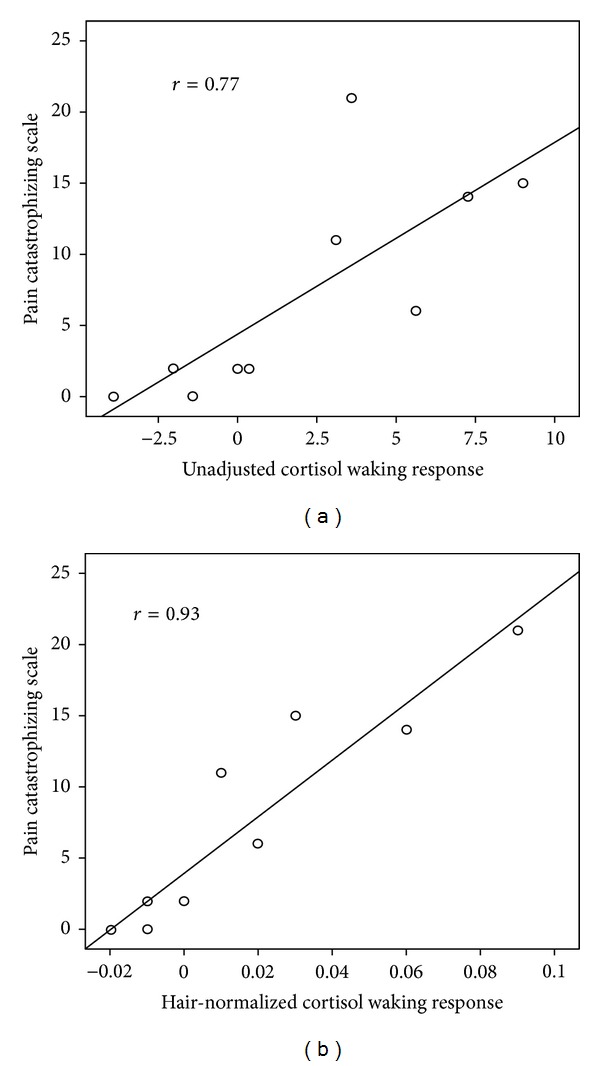
Scatterplot showing the relationship between unadjusted cortisol waking response (a) and hair-normalized cortisol waking response (b) with concurrent scores on the pain catastrophizing scale. Both correlations are significant at the *P* < 0.01 level.

Table 2 provides the results of the cross-sectional analyses at baseline. A strong and significant correlation was identified between baseline PCS score and both unadjusted CWR (*r* = 0.77, *P* < 0.01) and HnCWR (*r* = 0.93, *P* < 0.01; Figure 1) and a trend towards significance was identified for the relationship between baseline disability and HnCWR (*r* = 0.58, *P* = 0.08). No associations were significant for MDC or HnMDC.

**Figure 2:**
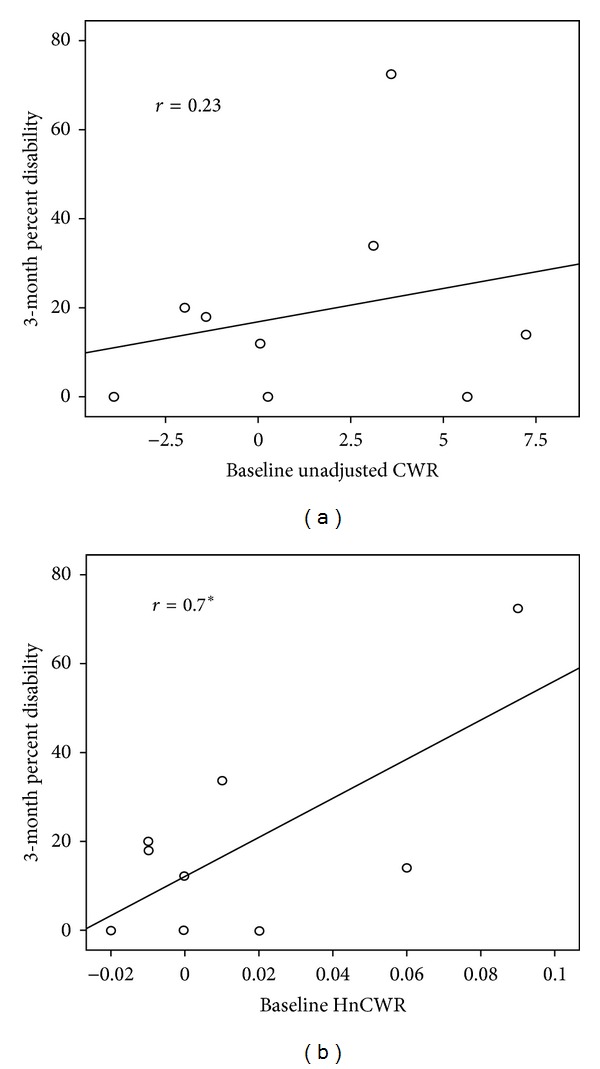
Scatterplot showing the relationship between unadjusted cortisol waking response (a) and hair-normalized cortisol waking response (b) with longitudinal (3 months) self-reported disability scores. * = correlation is significant at the *P* < 0.01 level.

**Figure 3:**
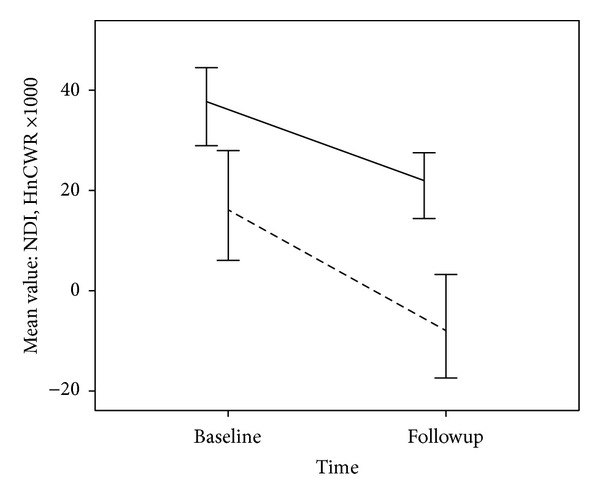
Mean neck disability index (NDI) scores at baseline (within 4 weeks of injury) and again at 3-month followup (solid line), plotted against hair-normalized cortisol waking response at the same time points (dashed line). HnCWR has been multiplied 1000 times in order to present both indicators on the same scale. Error bars represent 1 standard error of the mean (SE).

Table 3 provides the correlations between baseline (acute) cortisol variables and 3-month disability and pain intensity. Data of one additional subject were removed owing to clear evidence of data contamination at followup. With 9 remaining subjects, a significant positive correlation was found between HnCWR and 3-month disability (*r* = 0.70, *P* = 0.04; Figure 2). No other association was statistically significant.  Figure 3 plots the mean disability score at baseline and followup against the mean HnCWR (×1000) at the same time points. While longitudinal statistical modeling was inappropriate given the sample size, visually the figure suggests a parallel change in the two indicators over time.

## 5. Discussion

This proof-of-concept study suggests that normalizing the cortisol waking response for individual chronic cortisol secretion (as assessed by 3-month hair cortisol) may be of potential value in patients within the acute phase of posttraumatic injury. While the sample was too small to make definitive recommendations about changing clinical practice at this time, the magnitude of association with cross-sectional catastrophizing and longitudinal disability outcome suggest that HnCWR holds strong potential as a biomarker of distress in the acute phase of nonlife-threatening traumatic injury. The associations between nonnormalized CWR or MDC and the same cross-sectional or longitudinal variables were of trivial strength and were nonsignificant (not shown).

To our knowledge, this is the first time that state HPA activity has been evaluated as a function of trait activity for evaluation of acute posttraumatic distress. Of particular value is that hair cortisol presents an opportunity to “look backwards” in time, to estimate preinjury cortisol levels, and therefore to potentially provide an indication of preinjury emotional or physical distress. The ability to objectively capture preinjury variables in a valid way represents one of the largest existing barriers to estimating and understanding posttrauma recovery trajectory. Since the majority of such research necessarily begins after an injury has occurred, most attempts to quantify preinjury health status have used patient recall that is prone to bias. At best, preinjury medical records can be analyzed, but unless the subject has presented recently or regularly to their health care provider, such analyses are of questionable value.

Kirschbaum and colleagues [37] showed that hair cortisol can provide a valid retrospective calendar of HPA axis activity in pregnant women up to 6 months prior. Manenschijn and colleagues have used windows of 3 and 6 months to identify a relationship between hair cortisol and risk of cardiovascular events [38] and between cortisol and clinical indicators of hypercortisolism (waist circumference and waist-to-hip ratio) [39]. We chose a 3-month window (3 cm of hair) as the preinjury comparator partly because it made more clinical sense to evaluate more proximal cortisol activity and partly because subjects would be eligible with at least 3 cm of hair rather than 6 cm. While 3 months are a logical window, some may argue that a 6-month window would provide a more accurate representation of “trait” preinjury HPA activity, in so far as the effect of a single high-activity month would have less of an effect on the overall mean. However, the magnitude of the associations found so far support our decision of a 3-month focus, and requiring a minimum of 6 cm of hair almost guarantees a sample bias towards overrepresentation of females.

The strong association between HnCWR and PCS score can be viewed as lending support for validity to both tools. HnCWR, interpreted as the* relative* cortisol waking response in comparison to the 3-month preinjury average, appears to be strongly related to self-reported emotional distress as measured on the PCS. Catastrophizing as a construct is thought to be both a trait and a state of individual reactions to adversity [40, 41]. That is, evidence exists to suggest that some people may be classified in general as catastrophizers, but that most people will “catastrophize” in certain situations. It has been proposed that the mechanism explaining the association between pain and catastrophizing may be biological at least as much as it is psychological [42]. Finan and colleagues [43] have presented evidence to suggest that the association between pain and catastrophizing may be genetically mediated, with the catechol-o-methyltransferase (COMT) gene as a prime candidate to explain that association. COMT has also been presented as a candidate gene for explaining the magnitude of salivary cortisol increase in children following the Trier Social Stress Test Paradigm [44]. While it would be premature to draw a connection based on our findings, it is possible that a point of convergence exists between the fields of cognitive psychology, molecular biology, and endocrinology with COMT haplotypes affecting both the cognitive experience of pain and the physiological reaction. Multivariate models that include variables from each field should be further explored to better clarify these associations.

With specific regard to mechanisms, readers should be aware that an association as shown here is not adequate proof for cause and effect. The value of cortisol in hair and saliva has been explored under the assumption that an increased relative CWR ratio refers to a relative increase in central HPA axis activity. However, the field remains young, especially with respect to hair assays, and confirmatory remarks regarding mechanisms should be reserved at this time. An association between HPA axis activity and posttraumatic distress has long been known, evidenced by early reviews on the topic such as those of Yehuda and colleagues [45]. Of note, however, recent evidence indicates *suppression* of HPA axis activity rather than exaggeration in chronic posttraumatic stress disorder [46] and fibromyalgia [47] possibly suggesting that a reversal of HPA activity occurs during the transition from acute to chronic painful or posttraumatic conditions. It is also premature to confidently declare a direct association between hair cortisol and central or “systemic” cortisol in recognition of existing academic debate on the topic. On one hand, consistent evidence indicates a strong association between hair cortisol and clinical syndromes associated with systemic distress or hypercortisolism, including the PTSD studies mentioned previously [45] and the clinical course of Cushing's disease [48]. In the study of Thomson and colleagues in particular, resection of the pituitary adenoma led to measurable reductions in both plasma and hair cortisol, strongly suggesting a direct link between the two [48]. Conversely, some lines of evidence suggest that hair cortisol is not a valid indicator of chronic stress, at least when measured using radioactive cortisol markers in guinea pigs [49]. Others opine that the hair follicle and surrounding dermis and epidermis itself may have its own “peripheral” HPA axis that functions independently of the central axis, and it is the function of these peripheral axes that is being measured in hair. If this were the case, it may alter the way results from hair cortisol are interpreted [50, 51]. Interestingly, the report of Ito and colleagues [50] indicated that these peripheral HPA axes were under direct influence of cortisol releasing hormone (CRH), the production of which appears to be suppressed in patients with high exogenous cortisol [48] as would be expected in a normal functioning negative feedback loop. If the peripheral HPA axis theory was indeed correct, such patients should show lower hair cortisol as a result of high CRH, but in fact the opposite was reported by those authors [50]. The meaning of these diverging lines of evidence remains unresolved, but the results as presented here are not affected by this debate.

While the cross-sectional association is interesting and potentially valuable, the longitudinal association may have the greater clinical impact if the results can be replicated. Predicting posttraumatic outcomes especially identifying those at risk of prolonged recovery has become an active area of research in many musculoskeletal trauma conditions. In the field of acute whiplash alone, the pace and magnitude of research has stimulated no less than 5 systematic reviews or meta-analyses on the subject of prognosis within the past 10 years [9, 52–55]. To date, the most consistent predictors of poor outcome are high pain intensity and high self-rated disability at inception, with cognitive variables like catastrophizing and fear also showing consistent value. While potentially valuable for prediction, the greater value would come from understanding the mechanisms that drive these relationships, with the hope that novel intervention strategies could then be designed to mitigate the risk of chronic problems. If relative increase in HPA axis activity can find further support, then this may provide a novel target for therapy in those at risk individuals. This is a potentially exciting avenue for further research in this area.

A key limitation of this study that needs to be recognized is the limited sample size. With samples this small, a single outlier can dramatically affect the magnitude of the relationship. For this reason outliers were carefully identified and removed in order to provide a more accurate representation of the association. Furthermore, visual observation of Figures 1 and 2 reveals a reasonable distribution of scores across the continuum of each scale with no obvious outliers. However, it is still very possible that few additional subjects could dramatically affect this relationship. The strength of the relationships, especially that between the PCS and HnCWR, provides some degree of confidence in the association, but until more data are collected, this should be considered no more than proof-of-concept. In fact, one could argue that the association is too strong at this stage, certainly well beyond that expected. Further investigation in a larger sample will be required here.

An additional limitation is reflected in the protocol for saliva collection; subjects collected the samples independently at home not under the supervision of a member of the research team. This was intentional and is not uncommon in the field (e.g., [56]). This is done to remove the potentially confounding effect of the stress of going to work or the potential stress of being in the laboratory or clinic environment. However, this does increase the potential for noncompliance with the measurement protocol, and in fact the magnitude of the CWR in this pilot study is lower than what has been published previously suggesting that, perhaps, the initial saliva sample was not obtained immediately upon waking. As an attempt at some degree of standardization in this regard, subjects completed a simple self-report form at each collection interval, on which they indicated the time they awoke that day, the current time of the collection, and indicated by a check box that they had followed the protocol (no eating, drinking, exercise, or intercourse for 1 hour prior). While the times reported on those forms were in accordance with the protocol, this is by no means an absolute protection against deviation. However, it provided at least some degree of confidence that subjects were at least aware of the protocol and tried to follow it. Finally, there was some evidence of sample contamination that occurred in one subject at baseline and another at 3-month followup. We were unable to identify the source of the contamination, but this highlights the importance of strict adherence to protocol and sterile handling procedures. From a practical perspective, this type of sampling can be more challenging than self-report measures since some patients may not have adequate hair available and others may be reluctant to have hair cut from their head. These were certainly challenges experienced during recruitment and sampling. Given the strong relationships between biological and psychological reports in this study, further exploration of this relationship is imperative. Exploring alternate strategies to make the sampling more palatable to subjects, longitudinal designs to look at predictive validity in larger samples and feasibility of creating practical tests that could be used in the clinic will be needed before this proof-of-principle finding can be scaled up for widespread clinical utilization.

## 6. Conclusions

Preliminary evidence has been presented that suggests the cortisol waking response measured in the acute phase after musculoskeletal trauma may provide important insights into the mechanisms of concurrent distress and future recovery status, but only when normalized to 3-month hair cortisol. While small sample size exposes potential risk of over interpretation, the magnitude of the associations provides confidence in HnCWR as a promising avenue for further research.

## Figures and Tables

**Table 1:** Characteristics of the sample, including baseline independent variables.

Characteristics	Value
Sex (no. female, %)	7 (70%)
Age (mean years, range)	28 (21 to 59)
Days injury to inception (mean, range)	12 (7 to 20)
Type of injury (no., %)	
Whiplash	6 (60%)
Distal radius fracture	4 (40%)
Medicolegal status (no., %)	
No claim	6 (60%)
Worker's compensation	1 (10%)
Motor vehicle insurance	3 (30%)
Pain intensity (mean/10, range)	3.6 (0 to 7)
Disability (mean, range)	32% (0 to 90%)
Pain catastrophizing scale (mean/52, range)	7.3 (0 to 21)

**Table 2:** Simple bivariate associations between key independent variables and the two indicators of HPA axis activity measured in the acute stage of injury.

Categorical	Mean daily cortisol (ng/mL)	Cortisol waking response (ng/mL)	Hair-normalized mean daily cortisol	Hair-normalized cortisol waking response
	Mean (SD)	Mean (SD)	Mean (SD)	Mean (SD)
Sex				
Male (*n* = 3)	17.78 (8.28)	−1.95 (1.98)	0.10 (0.03)	−0.01 (0.01)
Female (*n* = 7)	19.58 (8.07)	3.92 (3.71)*	0.09 (0.01)	0.03 (0.04)
Type of injury				
WAD (*n* = 6)	16.46 (8.01)	2.14 (3.80)	0.08 (0.02)	0.01 (0.03)
DRF (*n* = 4)	22.90 (6.18)	2.19 (5.49)	0.11 (0.02)	0.03 (0.05)

Continuous	Mean daily cortisol (ng/mL)	Cortisol waking response (ng/mL)	Hair-normalized mean daily cortisol	Hair-normalized cortisol waking response
	*r* (*P*)	*r* (*P*)	*r* (*P*)	*r* (*P*)

Age	0.03 (0.93)	−0.17 (0.65)	0.20 (0.61)	0.10 (0.78)
NRS	0.26 (0.47)	0.28 (0.44)	0.41 (0.28)	0.36 (0.31)
%Disability	0.24 (0.50)	0.08 (0.84)	0.53 (0.15)	0.58 (0.08)
PCS	0.29 (0.41)	0.77 (<0.01)**	−0.24 (0.54)	0.93 (<0.01)**

*Difference between male and female is significant at the *P* < 0.05 level. **Correlation is significant at the *P* < 0.01 level. WAD: whiplash associated disorder, DRF: distal radius fracture, NRS: numeric rating scale for pain intensity, PCS: pain catastrophizing scale.

**Table 3:** Pearson's product-moment correlations between baseline (acute) cortisol variables and self-reported percent disability at 3-month followup.

	3-month percent disability	3-month pain intensity
Cortisol waking response	0.24 (0.53)	0.05 (0.88)
Hair-normalized cortisol waking response	**0.70 (0.04)**	0.23 (0.53)
Mean daily cortisol	0.19 (0.63)	−0.01 (0.99)
Hair-normalized mean daily cortisol	−0.09 (0.83)	0.25 (0.52)

The bolded values indicated statistically significant results.
